# *In Situ* Nanopressing: A General Approach to Robust Nanoparticles-Polymer Surface Structures

**DOI:** 10.1038/srep33494

**Published:** 2016-09-19

**Authors:** Xiaojie Zhang, Junhui He, Binbin Jin

**Affiliations:** 1 Functional Nanomaterials Laboratory, Center for Micro/Nanomaterials and Technology and Key Laboratory of Photochemical Conversion and Optoelectronic Materials, Technical Institute of Physics and Chemistry, Chinese Academy of Sciences, Zhongguancundonglu 29, Haidianqu, Beijing 100190, China; 2 University of Chinese Academy of Sciences, Beijing 100864, China

## Abstract

We report a novel, facile and general approach, *in situ* nanopressing, to integrate nanoparticles and polymers in a thin film configuration, where both nanoparticles exposure and film robustness are indispensable for applications. By simply pressing silica nanoparticles into a polymer thin film under an external force, we successfully attained a nanoparticles-polymer thin film, where the silica nanoparticles were partly embedded in the polymer thin film. The outstanding characteristic of easy-to-fabricate nanoparticles-polymer thin films combined the properties of both materials, giving excellent antireflective and antifogging properties, as well as enhanced the robustness of composite thin film. This *in situ* nanopressing may not only provide an alternative to meet the challenge of constructing mechanically robust nanoparticles-polymer thin films that require nanoparticles on the film surface, but also enrich the methodology to integrate nanoparticles and polymers.

As the use of nanoparticles provides a wide variability in properties and functions ranging from optical, electric, magnetic and mechanical to catalytic, the combination of polymers with nanoparticles, opens a wide playground where structures, functions and, ultimately, properties can be designed and tailored^1^,^2^,^3^. Nanoparticles-polymer thin films are a very important and efficient platform to realize the multifunction of nanoparticles and polymers. General methods to construct nanoparticles-polymer thin films include coating the mixture of nanoparticles and polymers, assembling polymer and nanoparticle layer-by-layer to attain multilayer structures, depositing a nanoparticles layer on top of a polymer layer towards double layer structures, and etc. For example, Mackay and coworkers obtained nanocomposites by spin-casting mixtures of nanoparticles and polymer, which demonstrated a fine control of entropic and enthalpic effects^4^. Shiratori^5^ and coworker constructed by layer-by-layer assembly poly(ethylene imine)(PEI)/SiO_2_ thin film with enhanced abrasion durability, where the loopy structure of PEI accounted for the enhancement of abrasion durability. He and coworker^6^ deposited a hollow silica nanoparticles layer on top of an antifogging polymer layer and obtained unusual antifogging antireflective thin films with the maximum transmittance of 98.9%. Nevertheless, the adhesion between the polymer layer and the nanoparticles layer was generally weak probably due to the small contact area between polymer and nanoparticle. Gao^7^ and coworkers constructed an antifogging antireflective composite film by first using an AAO mold to nanoimprint a polymer to attain a moth-eye structure followed by depositing silica nanoparticles by electron beam evaporation. Unfortunately, many efforts had to be spent in constructing special and reusable molds^8^,^9^, and there was no comments on the mechanical robustness of the composite film.

Many applications, such as in the optical, surface-enhanced Raman scattering, biosensing and electrocatalytic fields, generally require that nanoparticles must be located on the surface to realize their intrinsic functions. Besides, nanoparticles-polymer thin films must be mechanically robust for long-term applications. However, the above methods hardly meet the challenge of constructing mechanically robust nanoparticles-polymer films with nanoparticles on the surface.

Cells in nature work their own way out to enhance the robustness of their multifunctional structures. The fact that proteins are partly embedded in the cell membrane (Figure S1) inspires us to construct composite thin films where nanoparticles are partly embedded in polymer thin films via a novel technique called *in situ* nanopressing. By simply pressing silica nanoparticles into a polymer thin film under an external force, we succeeded in attaining a mechanically robust antireflective antifogging nanoparticles-polymer thin film where silica nanoparticles were partly embedded in the polymer thin film. This *in situ* nanopressing may provide an alternative to meet the challenge of constructing mechanically robust nanoparticles-polymer thin films that require nanoparticles on the film surface and enriches the methods to integrate nanoparticles and polymers.

## Results and Discussion

### Morphology and structure

**Figure 1:**
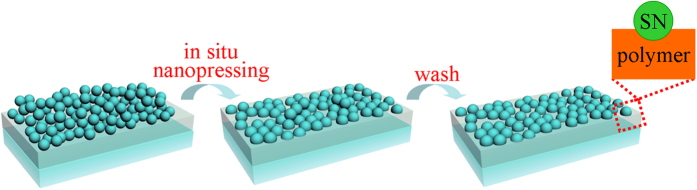
Schematic of *in situ* nanopressing process.

**Figure 2:**
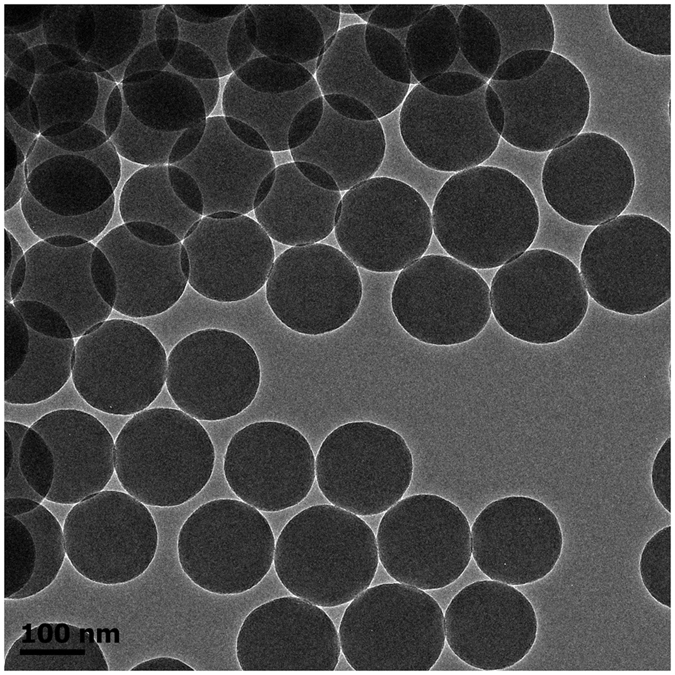
TEM image of silica nanoparticles (SNs).

**Figure 3:**
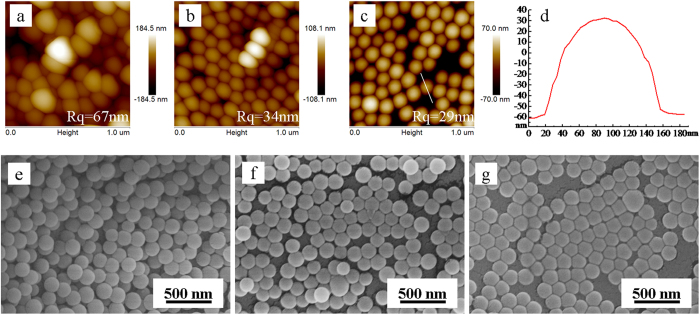
AFM images of (**a**) SNs/polymer/PET, (**b**) ISN-SNs/polymer/PET, and (**c**) ISNW20-SNs/polymer/PET, and (**d**) a height profile of ISNW20-SNs/polymer/PET along the selected line in AFM image (**c**); SEM images of (**e**) SNs/polymer/PET, (**f**) ISN-SNs/polymer/PET, and (**g**) ISNW20-SNs/polymer/PET.

“*In situ* nanopressing” in the current work represents “pressing existing components on nanoscale without additional template”. The procedure of *in situ* nanopressing is schematically illustrated in Fig. 1. Silica nanoparticles (SNs) which have been deposited on a polymer thin film are pressed into the polymer thin film under an external force. Those SNs which are not partly embedded in or attached firmly to the polymer thin film are eventually removed simply by washing the resulting specimen using a sponge. The Stöber method^10^ was employed to prepare SNs. Figure 2 shows the transmission electron microscopy (TEM) image of SNs. Clearly, the SNs are monodisperse nanospheres with an average diameter of ca. 147 nm. The whole fabrication procedure of composite thin films includes (1) coating a polymer thin film on polyethyleneterephthalate (PET), (2) depositing the SNs on the polymer thin film, (3) *in situ* nanopressing the deposited SNs into the polymer thin film and thermally cross-linking the polymer for 5 min at 130–150 °C, (4) washing the surface of specimen 20 times using a sponge to remove any SNs which were not partly embedded in or attached firmly to the polymer thin film, and (5) washing additional 100 times (totally 120 times) to test the mechanical stability. The specimens at different stages are named as polymer/PET, SNs/polymer/PET, ISN-SNs/polymer/PET, ISNW20-SNs/polymer/PET, and ISNW120-SNs/polymer/PET, respectively. The surface morphology and roughness of each coating were investigated by both atomic force microscopy (AFM) (Fig. 3a–d) and scanning electron microscopy (SEM) (Fig. 3e–g). The distortion of SNs shapes in Fig. 3a,b is probably attributed to the interaction between the tip of probe and the SNs. Electron microscopy observations are generally more reliable. Therefore, we provide the SEM images of SNs/polymer/PET, ISN-SNs/polymer/PET, ISNW20-SNs/polymer/PET respectively in Fig. 3. Clearly, the SNs are monodisperse from the large area views, agreeing well with the TEM image in Fig. 2.

The surface morphology and roughness of the coatings differ from each other. Before *in situ* nanopressing, the SNs were distributed in a loose and mutilayer manner on the polymer thin film with a roughness of 67 nm. After nanopressing, however, the SNs were distributed in a compact and almost single layer manner with a lowered roughness of 34 nm. After simply washing the specimen surface 20 times using a sponge, those SNs which were not attached firmly to or partly embedded in the polymer thin film were removed, resulting in a further lowered roughness of 29 nm. The heights of SNs were measured as typically shown in Fig. 3c,d. The average height of SNs is ca. 91 nm on the surface of ISNW20-SNs/polymer/PET. Considering the SNs average diameter of ca. 147 nm, the average depth of embedded parts would be ca. 56 nm. Such partial embedment could not only expose the SNs on the polymer thin film surface, but would also significantly enhance the robustness of composite thin film.

### Optical properties

**Figure 4:**
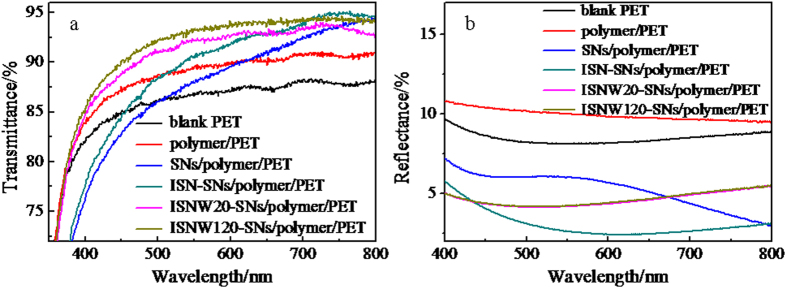
Transmission spectra (**a**) and Reflection spectra (**b**) of blank PET, polymer/PET, SNs/polymer/PET, ISN-SNs/polymer/PET, ISNW20-SNs/polymer/PET, and ISNW120-SNs/polymer/PET, respectively.

Figure 4 shows the transmission spectra and reflection spectra of blank PET, polymer/PET, SNs/polymer/PET, ISN-SNs/polymer/PET, ISNW20-SNs/polymer/PET, and ISNW120-SNs/polymer/PET, respectively. The polymer thin film not only increases the transmittance but also increases the reflectance as compared with blank PET. The SNs coating increases the transmittance in the wavelength range of 625–800 nm, but decreases the transmittance in the wavelength range of 400–625 nm, and significantly decreases the average reflectance by 4.7% in the whole wavelength range of 400–800 nm as compared with the polymer/PET. After *in situ* nanopressing, the ISN-SNs/polymer/PET has higher transmittance and lower reflectance than the SNs/polymer/PET in the wavelength range of 400–800 nm. After washing 20 times, the ISNW20-SNs/polymer/PET has higher transmittance in the wavelength range of 400–642 nm but lower transmittance in the wavelength range of 642–800 nm than the ISN-SNs/polymer/PET, and the average reflectance increases by 1.5% as compared with the ISN-SNs/polymer/PET. As summarized in Table 1, the maximum and average transmittance for the ISNW20-SNs/polymer/PET are 94.0% and 91.7%, and the minimum and average reflectance for the ISNW20-SNs/polymer/PET are 4.1% and 4.6% in the wavelength range of 400–800 nm, while blank PET has the maximum transmittance of 88.3%, the average transmittance of 86.7%, the minimum reflectance of 8.1% and the average reflectance of 8.5%, respectively, in the identical wavelength range. The partly embedded SNs have apparently brought the composite thin film the antireflective property, and have reduced the average reflectance by 3.9%.

Mechanical properties of thin films are a key issue, especially for outdoor applications. The mechanical properties of prepared thin films were assessed by washability. The thin film was washed using a sponge at a speed of 50 cycles per minute. If a thin film is not washed off and maintains the stable transmittance, it is believed to have good washability and can endure practical washing. In order to test the mechanical stability, we washed the surface of ISNW20-SNs/polymer/PET additional 100 times (totally 120 times) to test the mechanical stability. If the ISNW20-SNs/polymer/PET is mechanically robust, after additional washing 100 times, the transmittance and reflectance of ISNW120-SNs/polymer/PET should remain almost the same as those of ISNW20-SNs/polymer/PET. It is in fact the case. Even after extra washing 100 times (totally up to 120 times), the maximum and average transmittance for the ISNW120-SNs/polymer/PET remain as high as 94.7% and 92.7%, respectively, while the minimum and average reflectance remain as low as 4.2% and 4.7% in the wavelength range of 400–800 nm. Clearly, the transmittance and reflectance (Fig. 4a,b and Figure S2) remain nearly unchanged, indicating that the composite thin film is mechanically robust. The stable transmittance and mechanical robustness could be attributed to the enlarged contact area between the partly embedded SNs and the polymer, the hydrogen-bonding interaction, and the hexagonal close-pact assembly (Fig. 3c) created by in situ nanopressing. Therefore in situ nanopressing provides a simple approach to robust nanoparticles-polymer surface structures with the nanoparticles partially exposed on the surface.

### Antifogging property

**Figure 5:**
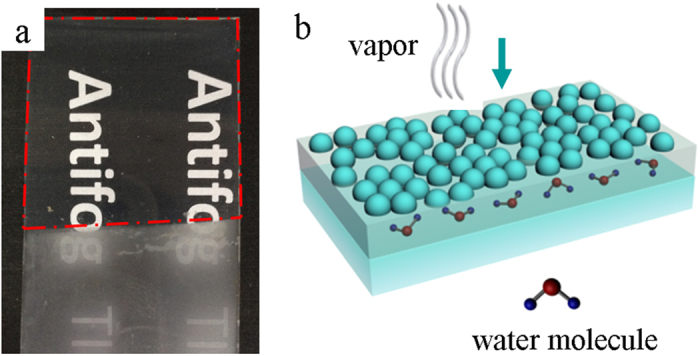
(**a**) Digital images exhibiting the antifogging property of blank (lower part) and ISNW20-SNs/polymer coated (upper part) PET, respectively; (**b**) Schematic illustration of the behavior of water vapour when encountering the ISNW20-SNs/polymer coated PET. Water vapour was absorbed into the polymer layer.

As shown in Fig. 5a, when the PET partly coated by the ISNW20-SNs/polymer thin film was cooled at −6 °C for 24 h in a refrigerator and then exposed to humid laboratory air (temperature: 20–30 °C, relative humidity: 20–40%), the uncoated part fogged immediately, the words below being blurred by strong light scattering of tiny water droplets. In contrast, the ISNW20-SNs/polymer coated part remained highly transparent, the words below being clearly visible. The water contact angle was 33.1° on the ISNW20-SNs/polymer/PET (Figure S3e). Therefore the antifogging property does not derive from the traditional superhydrophilic antifogging mechanism. As discussed in our recent works^6^,^11^, the antifogging property of ISNW20-SNs/polymer/PET derives from the hygroscopicity of the polymer layer although it is not located at the outmost surface. Figure 5b illustrates the behavior of vapour in the antifogging test when encountering the ISNW20-SNs/polymer coated PET. Water vapour was absorbed into the polymer layer. The polymer layer is like a reservoir or a sponge, and there is a sufficient volume to accommodate water molecules. The hygroscopicity of the polymer thin film is the intrinsic driving force while the voids, pores, and cracks in the SNs layer provide the passway for vapour, accounting for the excellent antifogging property. Therefore *in situ* nanopressing provides a general approach to integrate the properties of both materials. As nanoparticles and polymers used can be multiple, this *in situ* nanopressing technique would have great potential applications in antireflection, catalysis, biosensing, self-cleaning, surface-enhanced Raman scattering, electronic and optoelectronic fields.

## Conclusions

In conclusion, we have demonstrated a novel, facile and general approach, *in situ* nanopressing, to integrate nanoparticles and polymers in a thin film configuration, where both nanoparticles exposure and film robustness are required. The outstanding characteristic of easy-to-fabricate nanoparticles-polymer thin films is that the nanoparticles are partly embedded in the polymer thin film. This *in situ* nanopressing technique could combine the properties of both materials as well as enhance the robustness of composite thin film, and would thus meet the challenge of constructing mechanically robust nanoparticles-polymer thin films with the nanoparticles partly exposed on the thin film surface. As nanoparticle and polymer used can be multiple, this approach would have great potential in antireflection, catalysis, biosensing, self-cleaning, surface-enhanced Raman scattering, electronic and optoelectronic fields. The fine-controlled nanoparticles-polymer film configuration where the nanoparticles are partly embedded in the polymer would therefore largely expand the possibilities of materials design.

## Methods

### Chemicals

Poly(vinyl alcohol)(PVA, *M*_n_ = 70000–90000 g/mol, 99% hydrolyzed) was purchased from Aladdin. Poly (acrylic acid) (PAA, 53 wt% in water, *M*_w_ = 4000–7000 g/mol) was purchased from Shandong Heli water treatment company. Tetraethyl orthosilicate (TEOS, 98+%) was obtained from Alfa Aesar. Aqueous ammonia (25%) and absolute ethanol (99.5%) were purchased from Beihua Fine Chemicals. Ultrapure water with a resistivity higher than 18.2 MΩ · cm was used in all experiments, and was obtained from a three-stage Millipore Mill-Q Plus 185 purification system (Academic).

### Preparation of silica nanoparticles

95 mL absolute ethanol (99.5%), 5 mL ultrapure water and 5 mL aqueous ammonia (25%) were mixed in a round-bottomed flask with stirring. Then the flask was put into a 40 °C water bath. 3 mL TEOS was added in the mixture when the temperature became stable. After 12 h, silica nanoparticles were obtained^10^.

### Thin film preparation

Polymer thin films were deposited on substrates by one-step dip-coating followed by thermal cross-linking. First, PET was sonicated in water for at least 10 min, and then treated with oxygen plasma (84 W, 5 min) at an oxygen flow of 800 mL·min^−1^. The PET was then immersed in an aqueous solution containing PVA and PAA for 40 s, where the molar ratio of hydroxyl groups to carboxyl groups was set to 6:1∼15:1 (RH: 15%~40%), and withdrawn at a speed of 50 mm·min^−1^ from the solution. The polymer thin film was dried at room temperature. After the PET coated by the polymer was completely dry, the polymer covered PET was immersed in a silica nanoparticles sol for 10 s, and withdrawn 4 times at a speed of 200 mm·min^−1^ from the sol. Then, the silica nanoparticles were pressed into the polymer thin film under an external force by pincer pliers. Finally, The PET coated by *in situ* nanopressed SNs/polymer were thermally cross-linked at 130–150 °C for 5 min. After simply washing the specimen 20 times using a sponge, those silica nanoparticles which were not attached firmly to or partly embedded in the polymer thin film were removed.

### Characterization of thin films

Transmission electron microscopy (TEM) images were taken on a JEOL JEM-2100F transmission electron microscope at 200 kV. Samples for TEM observations were prepared by first dispersing silica nanoparticles in ethanol by ultrasonication, followed by dropping on holey carbon-coated copper grids and drying at room temperature. The morphology and roughness of coating surfaces were characterized by atomic force microscopy (AFM) on a MM8-SYS scanning probe microscope (Bruker AXR) and scanning electron microscopy (SEM) on a Hitachi S-4300 scanning electron microscope operated at 10 kV. Transmission spectra in the wavelength range of 330–800 nm were recorded using a TU-1901 spectrophotometer (Beijing Purkinje General Instrument Co.). Reflection spectra in the wavelength range of 400–800 nm were recorded on a Varian Cary 5000 UV/Vis-NIR spectrophotometer. Water contact angles on the surface of different specimens were measured at ambient temperature on a Kino SL200B3 automatic contact angle meter. For the examination of antifogging property, the ISNW20-SNs/polymer/PET was cooled at ca. −6 °C for 24 h in a refrigerator, and then exposed to humid laboratory air (temperature: 20–30 °C, relative humidity: 20–40%).

## Additional Information

**How to cite this article**: Zhang, X. *et al*. *In Situ* Nanopressing: A General Approach to Robust Nanoparticles-Polymer Surface Structures. *Sci. Rep.* **6**, 33494; doi: 10.1038/srep33494 (2016).

## Supplementary Material

Supplementary Information

## Figures and Tables

**Table 1:** Maximum transmittance (*T*_max_), average transmittance (*T*_ave_), minimum reflectance (*R*_min_) and average reflectance (*R*_ave_) in the wavelength range of 400–800 nm of blank PET, polymer/PET, SNs/polymer/PET, ISN-SNs/polymer/PET, ISNW20-SNs/polymer/PET, and ISNW120-SNs/polymer/PET, respectively.

	Blank PET	Polymer/PET	SNs/polymer/PET	ISN-SNs/polymer/PET	ISNW20-SNs/polymer/PET	ISNW120-SNs/polymer/PET
*T*_ave_	86.7%	89.3%	88.7%	90.5%	91.7%	92.7%
*T*_max_	88.3%	91.1%	94.5%	95.2%	94.0%	94.7%
*R*_ave_	8.5%	9.9%	5.2%	3.1%	4.6%	4.7%
*R*_min_	8.1%	9.5%	2.9%	2.4%	4.1%	4.2%
