# Supplementary material for: In Situ Nanopressing: A General Approach to Robust Nanoparticles-Polymer Surface Structures
Source: Sci Rep. 2016 Sep 19;6:33494. doi: 10.1038/srep33494 (PMC5027593; doi:10.1038/srep33494)
Supplement: Supplementary Information [file srep33494-s1.doc]

Supplementary Material for

**In Situ Nanopressing: A General Approach to Robust Nanoparticles-Polymer Surface Structures**

Xiaojie Zhang^1,2^, Junhui He^1^,*, Binbin Jin^1,2^

^1^ Functional Nanomaterials Laboratory, Center for Micro/Nanomaterials and Technology and Key Laboratory of Photochemical Conversion and Optoelectronic Materials, Technical Institute of Physics and Chemistry, Chinese Academy of Sciences, Zhongguancundonglu 29, Haidianqu, Beijing 100190, China.

^2^ University of Chinese Academy of Sciences, Beijing 100864, China.

* Correspondence and requests for material should be addressed to Junhui He (E-mail: jhhe@mail.ipc.ac.cn).


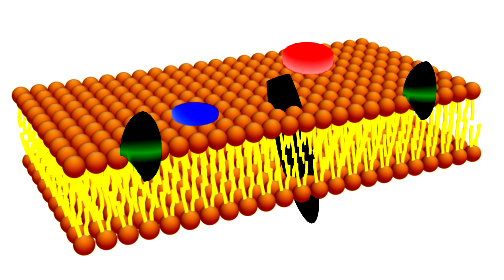


Figure S1 | The distribution of proteins on the cell membrane, the green one stands for a partially embedded protein on the cell membrane.

Figure S2 | Reflection spectra of ISNW20-SNs/polymer/PET and ISNW120-SNs/polymer/PET, respectively.

**
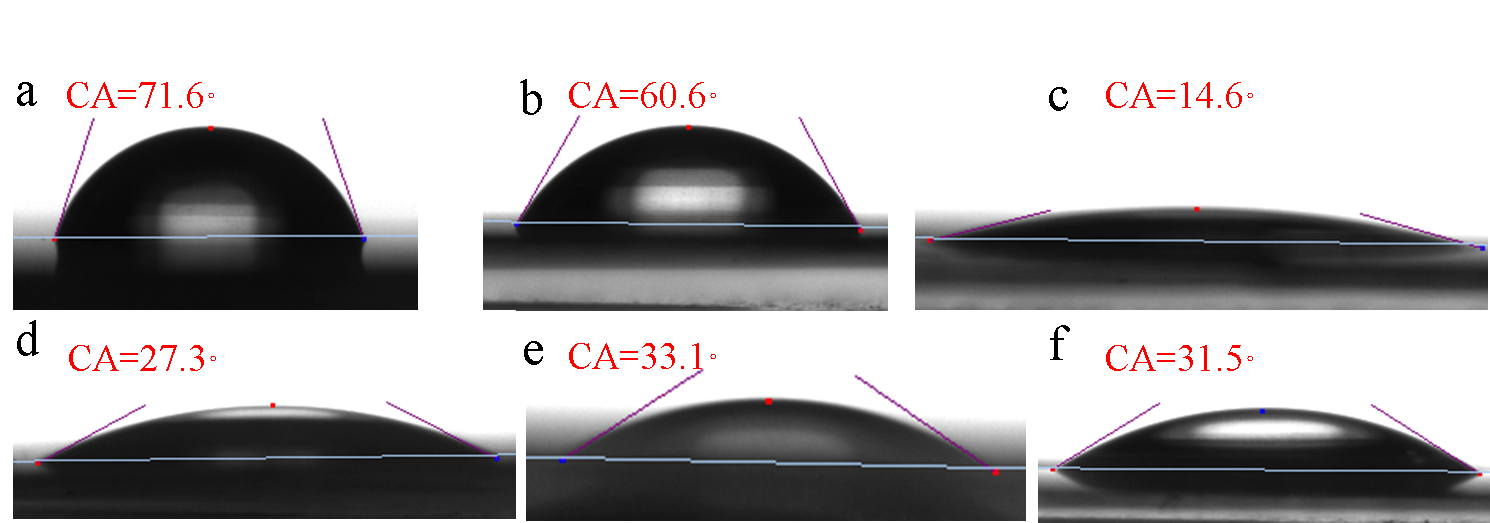
**

Figure S3 | Images of water contact angles on (a) blank PET, (b) polymer/PET, (c) SNs/polymer/PET, (d) ISN-SNs/polymer/PET, (e) ISNW20-SNs/polymer/PET, and (f) ISNW120-SNs/polymer/PET, respectively.
